# Biomechanical evaluation of cerclage wiring in plated tibia fractures using human and synthetic specimens

**DOI:** 10.1007/s00068-025-02894-8

**Published:** 2025-06-11

**Authors:** Sabrina Sandriesser, Stefan Förch, Jan Reuter, Christoph Kern, Marianne Hollensteiner, Edgar Mayr, Peter Augat

**Affiliations:** 1 Institute for Biomechanics, BG Unfallklinik Murnau, Prof. Küntscher Str. 8, 82418 Murnau, Germany; 2 Institute for Biomechanics, Paracelsus Medical University, Strubergasse 21, Salzburg, 5020 Austria; 3 Department of Trauma, Orthopaedic, Plastic and Hand Surgery, University Hospital of Augsburg, Stenglinstrasse 2, 86156 Augsburg, Germany

**Keywords:** Cable cerclage, Tibia, Synthetic bone, Biomechanics, Cerclage loosening, Incision

## Abstract

**Purpose:**

Supplemental cerclage wiring at the distal tibia has shown to improve the mechanical stabilization of the fracture. However, its clinical use remains controversial partly because of concerns about implant loosening as well as frictional or constrictive interference between the cerclage and cortical surface. The aim of this study was to investigate in a distal tibia fracture model possible loosening of the cerclage and interference with the bone surface.

**Methods:**

Fourteen distal tibia oblique fractures (AO/OTA 42-A2) in seven human and synthetic bone samples, respectively were treated by plate osteosynthesis with supplemental cerclage wiring. The samples were subjected to 50,000 load cycles under combined axial and torsional loads. Angular and axial displacement were continuously monitored to identify possible cerclage loosening cerclage. Potential incision of the cerclage was inspected visually and on CT scans.

**Results:**

Angular displacement was significantly influenced by the bone material (*p* ≤ 0.001) and type of osteosynthesis (*p* ≤ 0.001), while axial displacement was only influenced by the type of osteosynthesis (*p* ≤ 0.001). Lowest displacements were found in samples with plate and cable cerclage. Cerclage stability was maintained throughout the entire test period of 50,000 load cycles for human and synthetic samples. CT images revealed notches from the cerclage clamping mechanism but no incisions of the cable itself.

**Conclusion:**

In an oblique distal tibia fracture model, loosening of the cable cerclage was not detected. Full weight-bearing cyclic loads resulted in slight imprints of the cerclage crimp in both, human and synthetic samples. Following the surgical guidelines for careful cerclage installation, a supplemental cable cerclage has the potential to significantly increase the construct stability.

## Introduction

Cerclage wiring is a widely used surgical technique to enhance implant stability, especially in spiral or oblique fractures of long bones [1]. Cerclages are rarely applied as a stand-alone osteosynthesis, but are generally used in periprosthetic fracture management and in conjunction with plates or intramedullary nails [2–5]. The primary objective of supplemental cerclages is a reduction of interfragmentary shear motion, which is known to impede callus formation and consequently fracture healing [6, 7]. Extending the clinical and biomechanically safe use of cerclages, it has recently been demonstrated that supplemental cerclage wiring enhances the stability of the primary osteosynthesis in distal tibia spiral fractures [8–10]. Promising clinical results were found in a retrospective analysis of 96 tibia shaft spiral fractures with a total of 113 additive cerclages. Only three cases required material removal because of local soft tissue irritation from the cerclage [11].

Despite encouraging clinical and biomechanical findings, cerclage wiring at the distal tibia remains controversially discussed. Soft tissue damage, disruption of the periosteal blood supply due to cerclage incision and cerclage loosening are topics of continuous discussion [12–14]. From a clinical perspective, concerns that cerclage wiring could impair the blood supply were not confirmed [15]. No extended soft tissue trauma is anticipated when following the operative technique for minimally invasive cerclage application [11]. A biomechanical study by Lenz et al. revealed an intact cortical surface with only point contact of the cerclage fixation after a quasi-static load protocol up to 400 N [16]. Additional research is required to fully comprehend the biomechanical characteristics of cerclage wiring, as well as to bridge the knowledge gap regarding cerclage incision and possible loosening during full weight-bearing cyclic loads.

Most biomechanical investigations use either human bone samples or synthetic bone surrogates for a clinically relevant study design [17]. According to a study on screw cut-through behavior of intramedullary nails at the distal tibia, custom-made synthetic bone surrogates based on polyurethane realistically replicate the properties of human bone [18]. Currently, no validated synthetic bone samples are available in terms of cerclage incision into the cortical bone and possible cerclage loosening.

The aim of this biomechanical study was to investigate the interaction of cable cerclages with the cortical shell in oblique fractures of the distal tibia that were primarily stabilized by angle stable plate osteosynthesis. Possible cerclage loosening and incision into the bone under full weight-bearing cyclic loading were analyzed. Additionally, custom-made synthetic bone samples were validated against human distal tibia samples. The experiments were repeated for solitary plate osteosynthesis to demonstrate the increased stabilizing effect of supplemental cable cerclage wiring. The following hypotheses were postulated: (1) Cable cerclages do not loosen under full weight-bearing cyclic loads. (2) Cable cerclages do not cut into cortical bone under full weight-bearing cyclic loads. (3) Custom-made synthetic bone surrogates can replicate the mechanical characteristics of human bones. (4) Supplemental cable cerclages stabilize the osteosynthesis construct compared to solitary plate osteosynthesis.

## Materials and methods

In this study a total of fourteen samples were tested. Seven fresh frozen human distal tibiae from one male and six female donors at a mean age of 45 ± 7 years were included. Seven polyurethane based custom-made synthetic bone surrogates of the distal tibia were manufactured, including an intramedullary cavity at a diameter of 8 mm. The material composition was identical to that of a distal tibia screw cut-out study on custom-made synthetic bones [18].

### Specimen preparation

**Fig. 1:**
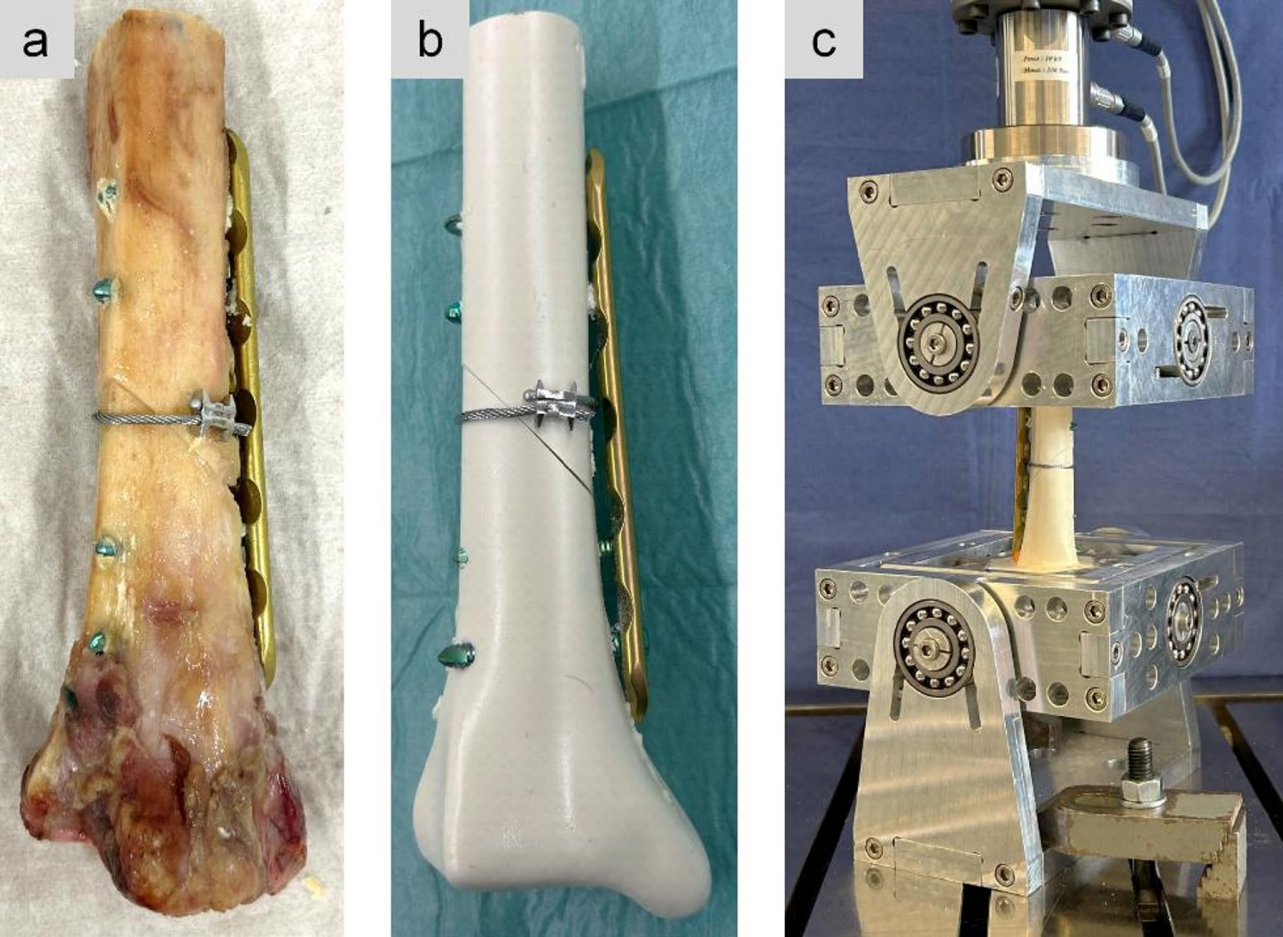
Oblique fracture at the distal tibia stabilized by plate osteosynthesis and supplemental cable cerclage wiring in human bone (**a**) and custom-made synthetic bone (**b**). Test setup with two cardan joints to reduce constraint forces (**c**)

The human specimens were thawed and remaining soft tissue was dissected. The human bones were kept moist using a physiologic saline solution. Both, human and synthetic bones were cut to a length of 15 cm. First, to simulate a 45° oblique fracture (AO/OTA 42-A2), an osteotomy was made with the fracture line from proximal-lateral to distal-medial. A vice was used to keep the two fragments in place and to achieve fracture reposition. In a next step, a steel cable cerclage (ø 1.7 mm, 298.801.01, DePuy Synthes, Johnson & Johnson) was looped around the fracture zone and tensioned at 500 N according to the manufacturer’s recommendation. Finally, locking plate osteosynthesis (4.5/5.0 LCP, narrow, 6-hole, Titanium 424.561, DePuy Synthes, Johnson & Johnson) with two screws per fragment was applied and tightened at 4 Nm (Fig. 1). All implantations were performed by an experienced trauma surgeon.

## Mechanical setup

The samples were proximally and distally embedded for 4 cm in a three-component casting resin that cured into a rigid polyurethane material (Fast Cast Resin 010 A/B + Filler DT 082 − 1, Goessl + Pfaff GmbH, Karlskron, Germany). Modeling clay was applied to the plate and the screw tips to prevent the implant from embedding. The samples were mounted in an electrodynamic testing machine (Zwick LTM 10 T, ZwickRoell GmbH & Co. KG, Ulm, Germany) with two cardan joints to reduce constraint forces. Throughout testing, the human bones were kept moist with gauze swabs that were sprayed with saline solution.

Combined axial and torsional full weight-bearing loads at an average body weight of 75 kg were applied to simulate physiological cyclic loading of the tibia. Axial sinusoidal load ranging from 50 N to 750 N was set at a frequency of 2 Hz. Torsional loads were applied alternately to + 4 Nm and − 4 Nm at a frequency of 1 Hz. 50,000 load cycles were applied to approximate the number of steps taken by patients during the first four weeks after surgery [19]. In order to investigate the stabilizing effect of the additional cable cerclage, the samples were not loaded to failure but were tested within the elastic limits of the plate. After testing the plate and cable cerclage construct (Plate + Cable), the cerclage was removed and tests were repeated with the solitary plate osteosynthesis (PlateOnly).

### Data analysis

In order to enable 200 settling cycles and guarantee efficient data compaction, machine data was examined at three distinct time points: at the beginning (200th cycle), halfway through the test (24,000th cycle) and before test termination (49,000th cycle). Angular displacement was defined as the difference in angulation between the minimum (−4 Nm) and maximum (+ 4 Nm) applied torque for each of the three time points, respectively. Axial displacement was defined as the difference in displacement between the minimum (50 N) and maximum (750 N) applied load for each of the three time points, respectively. Loosening of the cable cerclage can be assumed if the displacement increases significantly over the applied load cycles. The applied torque and the angular displacement were used to calculate torsional construct stiffness. Furthermore, permanent deformation was calculated as the difference in movement between the 200th and 49,000th cycle for angular and axial displacement, respectively.

Following mechanical testing, the explanted samples were CT scanned (Siemens Somatom) with a slice thickness of 0.2 mm in order to analyze any possible incision of the cerclage. Pictures were taken to document visual inspection.

Statistical tests including boxplots were performed using SPSS (version 26, IBM SPSS Statistics, NY, US). Normal distribution of the data was tested with Shapiro-Wilk test and homogeneity of variances was tested with Levene’s test. The impact of the bone material (human vs. synthetic) and the type of osteosynthesis (Plate + Cable vs. PlateOnly) were examined using a two-way ANOVA. Differences in the bone material were further analyzed with unpaired t-tests. The effect of the osteosynthesis was further analyzed with paired t-tests. Additionally, one-way ANOVAs were used to analyze the effect of the time point for each group separately. In one human PlateOnly sample implant failure due to screw breakage terminated the test after 24,000 cycles. This sample was omitted from data analysis for the last time point after 49,000 cycles. In the boxplots the values are displayed as median and the interquartile range, while in the text mean ± standard deviation is provided. Statistical analysis was conducted using a significance level of 0.05.

## Results

Torsional construct stiffness was significantly higher for the human bone with plate and cable cerclage (8.4 ± 5.2 Nm/°) compared to all other test groups (Human_PlateOnly 2.3 ± 1.4 Nm/°, Synthetic_Plate + Cable 2.8 ± 0.1 Nm/°, Synthetic_PlateOnly 1.6 ± 0.2 Nm/°, *p* < 0.01 each).

**Fig. 2:**
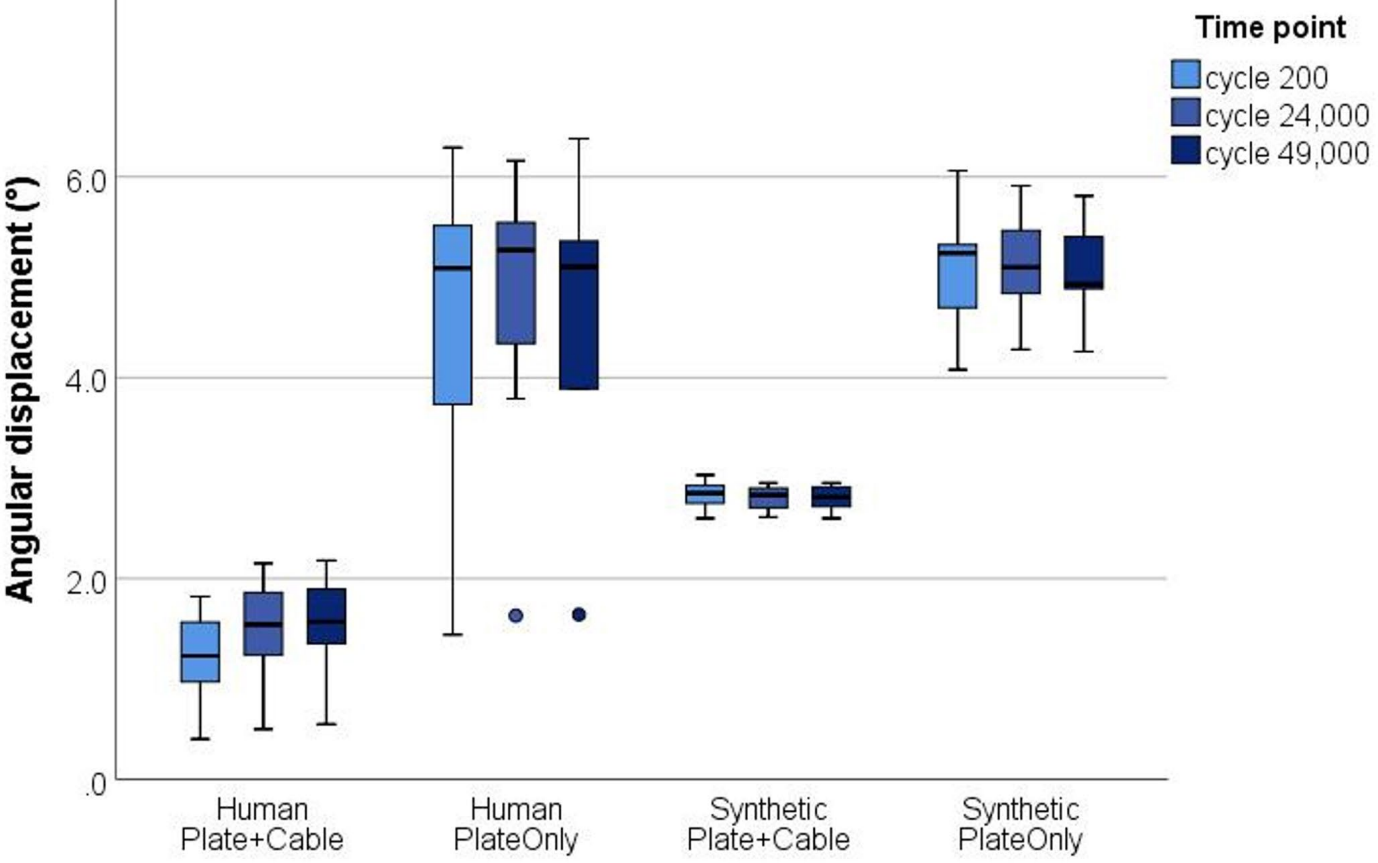
Angular displacement (°) at the three measured time points (200th, 24,000th and 49,000th cycle) of human and synthetic samples. Dots represent outliers beyond 1.5xIQR (interquartile range)

Two-way ANOVA revealed that angular displacement was significantly influenced by the kind of bone material (*p* ≤ 0.001) and type of osteosynthesis (*p* ≤ 0.001) (Fig. 2). Lowest angular displacement of about 1.5 ± 0.5 ° was measured for the human bone with plate and cable cerclage. In synthetic bone samples with plate and cable cerclage movement nearly doubled to 2.8 ± 0.1 ° (*p* ≤ 0.001). Both bone materials in the PlateOnly groups showed comparable movement (*p* = 0.416). Without additional cerclage wiring, angular displacement significantly increased to 4.6 ± 1.5 ° for human (*p* = 0.001) and 5.1 ± 0.5 ° for synthetic bone samples (*p* ≤ 0.001). Permanent angular deformation after cyclic testing was below 0.4 ° for both human groups and remained negligible small (0.03 °) for both synthetic groups. Thus, no significant loosening of the cerclage was observed in any of the cable cerclage groups (*p* > 0.51, respectively).

**Fig. 3:**
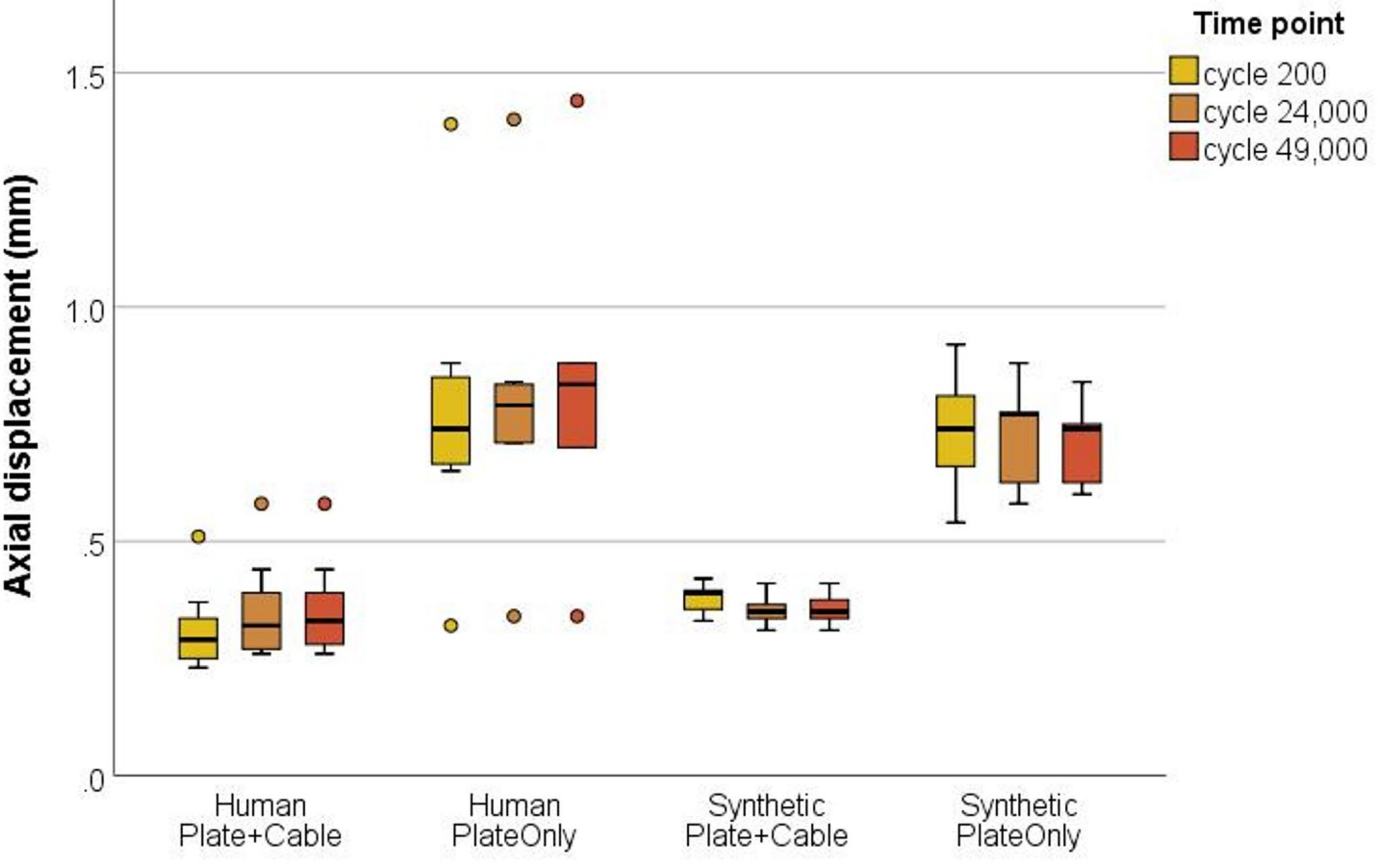
Axial displacement (mm) at the three measured time points (200th, 24,000th and 49,000th cycle) of human and synthetic samples. Dots represent outliers beyond 1.5xIQR (interquartile range)

Two-way ANOVA revealed that axial displacement was significantly influenced by type of osteosynthesis (*p* ≤ 0.001), but the type of bone material revealed comparable displacements (*p* = 0.371) (Fig. 3). Lowest axial displacement was measured for both groups receiving plate and cable cerclage (human 0.3 ± 0.1 mm, synthetic 0.4 ± 0.1 mm, *p* = 0.136). Both bone materials in the PlateOnly groups showed comparable movement (*p* = 0.718). Without additional cerclage wiring, axial displacement significantly increased to 0.8 ± 0.3 mm for human (*p* = 0.003) and 0.7 ± 0.1 mm for synthetic bone samples (*p* ≤ 0.001). Permanent axial deformation after cyclic testing was below 0.08 mm for both human groups and negligible small (0.03 mm) for both synthetic groups. Thus, no significant loosening of the cerclage was observed in any of the cable cerclage groups (*p* > 0.36, respectively).

**Fig. 4:**
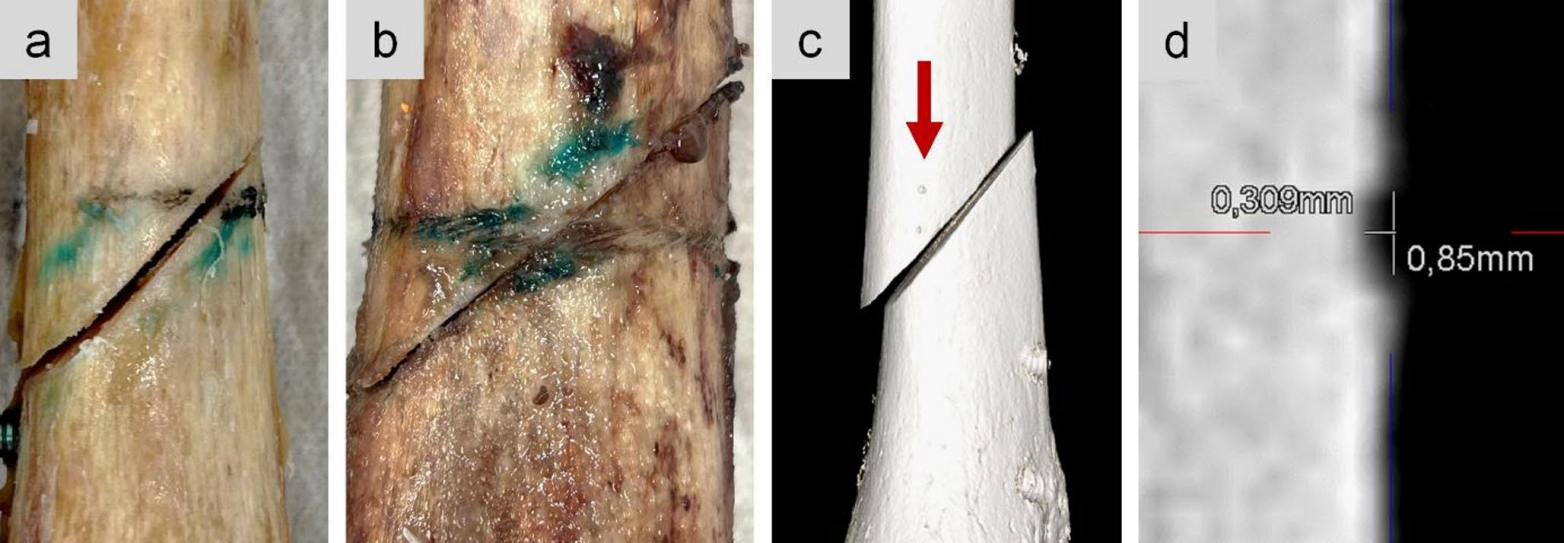
Visual inspection of cerclage imprints in a human sample (**a**, **b**). The blue staining resulted from manual marks used to create the oblique fracture. In CT scans only the notches from the cerclage clamping were visible (**c**). Exemplary measurement of the notch size in human bone (**d**)

**Fig. 5:**
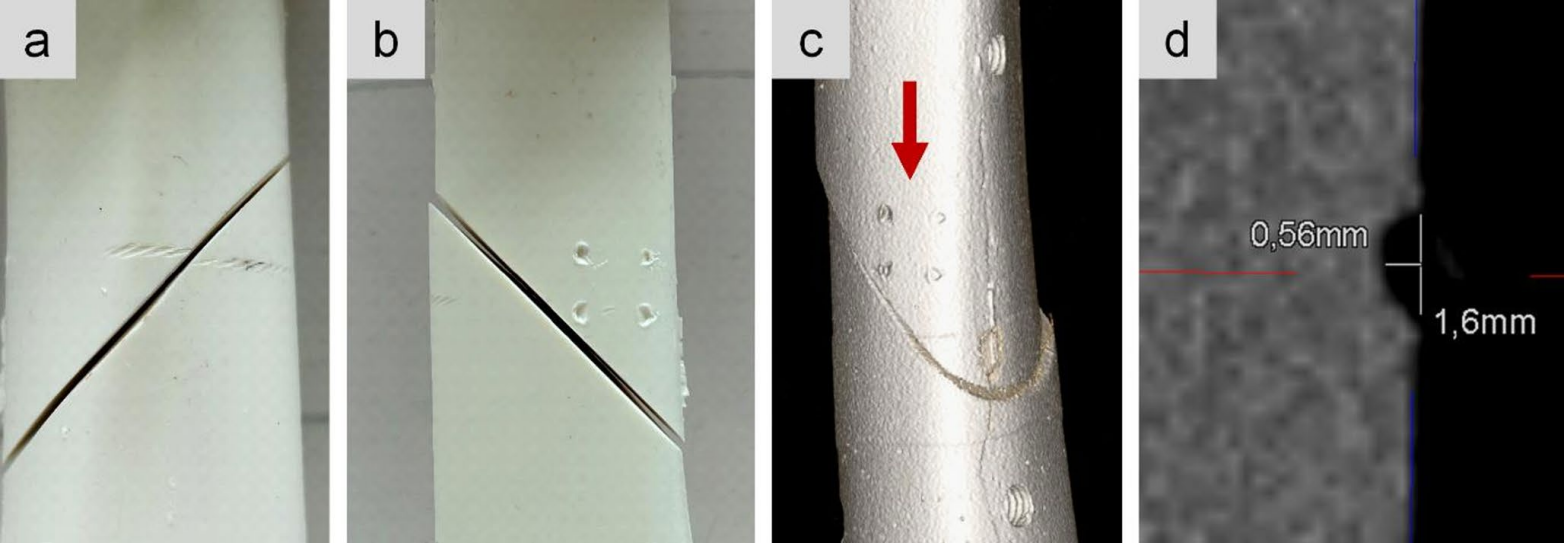
Visual inspection of cerclage imprints (**a**) and notches from the cerclage clamping (**b**) in a synthetic sample. In CT scans only the notches from the cerclage clamping were visible (**c**). Exemplary measurement of the notch size in synthetic bone (**d**)

Only small notches caused by the four pins of the cerclage clamping mechanism were seen on CT scans with a slice thickness of 0.2 mm (Figs. 4 and 5). Those notches were 0.85 mm in the human samples, compared to 1.6 mm in the synthetic samples. Visual inspection identified superficial incisions from the cable cerclage, which were not detectable on CT scans. With the bare eye both, the human and synthetic bones, revealed small imprints of the braided cable cerclage. The imprints were not circumferential as the geometry of the tibia shaft is not circular.

## Discussion

This biomechanical study provided further insights on the interaction of cable cerclages with the cortical bone in a distal tibia fracture model. Since supplemental cerclage wiring at the distal tibia remains controversially discussed, this study focused on clinically relevant characteristics of cable cerclages that can be examined within a biomechanical investigation. Our study revealed no evidence of cerclage loosening or circumferential incisions during full weight-bearing cyclic loads.

Cerclage loosening and initial tension loss may be influenced by different factors. Initial tension loss was found to be present at various cerclages from different manufacturers. The crimp mechanism, which was used in this present study, was found to outperform in terms of initial tension loss [20]. In an insufficiently reduced fracture the induced motion can facilitate cerclage loosening and migration and thus induce necrosis [1, 16]. Furthermore, loosening can be attributed to the type of locking system, the use of crimps or set screw clamps, postoperative micro-motion or to the soft tissue condition [20].

Biomechanical properties of different cerclage types and materials as well as their interaction with cortical bone have been addressed in various literature [16, 21–24]. Still, in a distal tibia fracture model cable cerclage loosening and incision due to cyclic loading, which seems to be the decisive factor for loosening, have not been adequately assessed so far. A comparable study on distal tibia fractures did not observe any loosening after cyclic testing; however, loosening was not quantitatively analyzed but only visually inspected [8]. Hägerich et al. loaded simplified constructs of cortical half-shells in a rapid cyclic ramped test to 1000 N and found that a titanium cable cerclage provided higher compression force onto the bone than steel wire cerclages or suture tapes [21]. Our study seems to be the first biomechanical investigation to assess cerclage loosening in a relevant fracture model. We applied a reasonable load protocol of 50,000 cycles to simulate the first four weeks after surgery and thus the early phase of bone healing [19]. During cyclic loading, we found no cerclage loosening in either human or synthetic bone samples.

To minimize incisions at the cerclage-bone interface, cerclages should be installed carefully by following the surgical guidelines [11]. Especially in poor bone quality, the cerclage tightening process should be monitored by respective tensioning devices to reduce the risk of high radial forces on the cortical bone [21]. In this study, the cerclage was tensioned at 500 N according to the manufacturer’s recommendations. In a study by Lenz et al., it was shown on femoral shaft models that cortical bone is stable enough to withstand radial forces of the cable cerclage without damaging the underlying cortical surface [16]. Histological evaluation of the loaded cortical areas underneath the cerclage showed an intact bone without damaged cortical surface. Additionally, the stress distribution on the femoral shaft was examined using pressure films, which revealed only point contacts by the cerclage. Moreover, in axial view the femoral geometry is not perfectly circular. Thus, the cerclage only exhibits point contacts and does not circumferentially compress the periosteum [16]. It has to be investigated in further studies, whether more flexible cerclages circumferentially compress the cortical bone. Due to a triangular shaped diaphysis, only point contacts by a cable cerclage can be presumed for the tibia as well. The results of the current study support the assumption that cable cerclages do not cause circumferential incisions. In all human and synthetic samples, the braided imprints of the cable cerclage were solely point contacts rather than circumferential. More distinct incisions were caused by the four “teeth” of the crimp fixation. For human samples it was not possible to visually distinguish whether the cerclage cut into the cortical surface or the braided imprints were solely in the remaining soft tissue and periosteum. The braided imprints were not detected in CT scans of either human or synthetic samples. The resolution of the clinical CT was limited to a slice thickness of 0.2 mm. For future investigations micro-CT scans with a higher resolution should be taken into consideration. A weakness of the study is that it was not examined if the imprint and the notches were caused under cyclic load or if they were already present after cerclage tightening at 500 N.

Using supplemental cerclages raises further concerns about the cerclage-bone interface, including the potential for soft tissue damage and the associated risk of disrupted periosteal blood vessels. Various studies have investigated the feared impairment on blood supply caused by supplemental cerclage wiring at the tibia [2, 15]. Especially at the distal tibia, the scarcity of soft tissue may discourage the use of cerclages. Following a minimally invasive surgical approach with careful soft tissue management, a retrospective study on 96 tibia shaft spiral fractures treated by supplemental cerclage wiring revealed no evidence of extended soft tissue trauma [11]. Only three cases (3%) revealed local irritation due to cerclage wiring. No increased risk of vascular injuries or neurological damage caused by cerclage wiring was observed. The cerclage lock was placed on the dorsomedial edge due to a more robust soft tissue condition. Although all soft tissue has been resected from the specimens in our study, we followed this recommendation and placed the crimp on the dorsomedial edge of the tibia.

In the current study only the 1.7 mm steel cable cerclage was examined. Supplemental steel cable cerclages provide the highest stability when different cerclage materials were compared [9]. Orthopaedic cerclages for fracture fixation generally consist of stainless-steel or titanium cables, wires, or non-metallic cerclages, such as suture tapes. Especially for suture tapes, recent studies investigated the optimal knot technique and their biomechanical performance [21, 22, 25, 26]. Non-metallic material eliminates the risk of metal-related allergies and metallosis. Whether suture tapes exhibit similar biomechanical results under cyclic loading or if they loosen under physiologic load scenarios has to be investigated in future studies.

The stabilizing effect of supplemental cerclage wiring in distal tibia spiral fractures has already been investigated in biomechanical studies [8–10]. The current study supported these findings. Solitary plate osteosynthesis resulted in significantly increased angular and axial displacements for both, human and synthetic bones. A limitation of this study includes the reduced primary stability by a large fragment 6-hole locking plate. However, this plate was selected to provoke a worst-case scenario for the cable cerclage. Usually, this type of fracture is treated by a metaphyseal locking compression plate or, in case of a more proximal shaft fracture, by intramedullary nailing.

Various cerclage performance studies solely focused on the type of cerclage, thus tolerating simplified and non-physiological setups [16, 21, 23, 24]. In recent biomechanical studies, clinically relevant distal tibia fracture models were tested under physiologically combined axial and torsional loads [8–10]. We adopted the combined axial and torsional load protocol and applied full weight-bearing loads at an average body weight of 75 kg. Full weight-bearing seemed appropriate to cover the majority of post-operative rehabilitation exercises. Generally, knee joint loads range from partial to full body weight, and during daily activities like walking, ascending or descending stairs, or stand up and sit down from a chair, the load can increase to three times the body weight [27].

Immediate weight-bearing as tolerated seems to gain attention in postoperative rehabilitation, especially to promote early mobilization in geriatric patients [28–30]. Two randomized controlled trials independently found that full weight-bearing appeared to be beneficial for healing and fastens the recovery process without raising the risk of complications [31, 32]. Although recent literature proves that augmentation of the osteosynthesis by cerclage wiring allows for immediate weight-bearing as tolerated, postoperative mobilization after oblique or spiral fractures of the tibia remains often restricted to partial weight-bearing [11].

In order to justify the use of custom-made synthetic bone surrogates for the purpose of this study, the use of bone surrogates was validated with fresh frozen human bone specimens. For experimental testing, bone surrogates are intended to ideally replicate the morphological and biomechanical properties of human bones, while avoiding the inherent drawbacks, such as possible infection risk, population variability and increased procurement and storage costs [17]. The polyurethane based synthetic material provided promising results when investigating the cut-out behavior of locking screws in distal tibia nails [18]. In the current study the same material composition was used to form anatomically correct models of the distal tibia. For solitary plate osteosynthesis, the synthetic bone surrogates realistically mimic the properties of human distal tibiae in an oblique fracture model under full weight-bearing loads. The synthetic samples only allowed higher angular displacement when the construct stability was increased by supplemental cerclage wiring. In terms of torsional stiffness, synthetic bones were less stiff than the human tibiae. This could explain the fact that the four notches from the cerclage clamping were more distinct in synthetic bone. Braided imprints of the cable cerclage were visually detected in both, human and synthetic bones. Recently, custom-made synthetic bones have the capability to represent the biomechanical properties of human bone and address its population variability [33]. Custom-made synthetic femora, for example, provide also osteoporotic models and already realistically replicate human femora in terms of axial compression, bending, and torsional stiffness [34]. However, to fully replicate the characteristics of human distal tibia cortical bone, the material composition should be adjusted to provide an even harder cortical shell.

In addition to the aforementioned constraints of the study further limitations need to be indicated. Due to limited availability of human specimens, we included six female samples and one male sample with an average donor age of 45 ± 7 years in this study. An evenly distributed study group, in terms of gender and age, might enhance the transferability into the clinical setting. Moreover, the same constructs have been used to repeat the tests with solitary plate osteosynthesis. This poses a risk of measurement bias. Finally, the application of supplemental cerclages generally remains limited to oblique or spiral fractures.

In conclusion, cable cerclage loosening in this specific fracture model at the distal tibia was not detected. The stability provided by the cerclage was maintained during the entire load cycles, which corresponds to a period of four weeks postoperatively. Full weight-bearing cyclic loads caused notches of the cerclage crimp and small imprints of the braided cable. However, the cable exhibits only point contacts to the cortical bone. Our results are consistent with promising clinical findings that a supplemental cable cerclage significantly increases the overall implant stability when installed carefully and in accordance with the surgical guidelines.

## Data Availability

No datasets were generated or analysed during the current study.
